# CHILDHOOD AND MIDLIFE CULTURAL ENGAGEMENT AMONG KOREAN MARRIED COUPLES

**DOI:** 10.1093/geroni/igac059.767

**Published:** 2022-12-20

**Authors:** Kyungmin Kim, Jeffrey Burr, Gyounghae Han, Bon Kim

**Affiliations:** Seoul National University, Seoul, Seoul-t'ukpyolsi, Republic of Korea; University of Massachusetts Boston, Boston, Massachusetts, United States; Seoul National University, Seoul, Seoul-t'ukpyolsi, Republic of Korea; Jeju National University, Seoul, Gyeonggi, Republic of Korea

## Abstract

Cultural reproduction theory posits that cultural resources transmit to the next generation, suggesting a lingering effect of parental influences on cultural experiences in adulthood. Further, middle-aged adults’ cultural engagement may not only be influenced by their own childhood experiences but also their spouses’ experiences. This study extends our understanding of childhood and midlife cultural engagement of married couples, using a sample of 1,271 couples (age 49–66) from the 2012 Korean Baby Boomer Panel Study and Korean Forgotten Generation Study. Results from Actor Partner Interdependence Models showed that beyond one’s own childhood cultural engagement, spouse’s childhood cultural engagement was associated with levels of perceived cultural engagement at midlife (for both husbands and wives) and number of arts and cultural activities at midlife (only for husbands). Given the cross-spousal associations in cultural engagement among Korean middle-aged couples, both spouses’ cultural resources need to be considered for policy implications.

